# Effect of Selenium on the Iron Homeostasis and Oxidative Damage in Brain and Liver of Mice

**DOI:** 10.3390/antiox11071216

**Published:** 2022-06-21

**Authors:** Inga Staneviciene, Jurgita Sulinskiene, Ilona Sadauskiene, Arunas Liekis, Ausrine Ruzgaite, Rima Naginiene, Dale Baranauskiene, Vaida Simakauskiene, Raulas Krusnauskas, Dale Viezeliene

**Affiliations:** 1 Department of Biochemistry, Medical Academy, Lithuanian University of Health Sciences, A. Mickeviciaus St. 9, LT-44307 Kaunas, Lithuania; jurgita.sulinskiene@lsmuni.lt (J.S.); ilona.sadauskiene@lsmuni.lt (I.S.); ausrine.ruzgaite@stud.lsmuni.lt (A.R.); dale.viezeliene@lsmuni.lt (D.V.); 2 Neuroscience Institute, Lithuanian University of Health Sciences, Eiveniu St. 4, LT-50161 Kaunas, Lithuania; arunas.liekis@lsmuni.lt (A.L.); rima.naginiene@lsmuni.lt (R.N.); dale.baranauskiene@lsmuni.lt (D.B.); vaida.simakauskiene@lsmuni.lt (V.S.); raulas.krusnauskas@lsmuni.lt (R.K.)

**Keywords:** selenium, iron, lipid peroxidation, catalase

## Abstract

Selenium is an essential trace element that maintains normal brain function, mainly due its antioxidant properties. Although the amount of Se in the body is tightly regulated by the liver, both an excess of and deficiency in Se can modulate the cellular redox status and affect the homeostasis of other essential elements for both humans and animals. The aim of this study was to determine the effect of inorganic selenium excess on oxidative stress and iron homeostasis in brain and liver of laboratory BALB/c mice, which were supplemented with Na_2_SeO_3_ solution (0.2 mg and 0.4 mg Se/kg body weight) for 8 weeks. The content of the lipid peroxidation product malondialdehyde and antioxidant enzyme catalase activity/gene expression were used as markers of oxidative damage and were evaluated by spectrophotometric assays. Selenium and iron concentrations were determined by inductively coupled plasma mass spectrometry (ICP-MS). Catalase gene expression was analyzed by qRT-PCR and ΔΔCt methods. Our results showed that doses of 0.2 mg Se and 0.4 mg Se caused a relatively low accumulation of Se in the brain of mice; however, it induced a 10-fold increase in its accumulation in the liver and also increased iron accumulation in both tested organs. Both doses of Se increased the content of malondialdehyde as well as decreased catalase activity in the liver, while the 0.4 mg Se dose has also activated catalase gene expression. Brain of mice exposed to 0.2 mg Se showed reduced lipid peroxidation; however, the exposure to 0.4 mg of Se increased the catalase activity as well as gene expression. One may conclude that exposure to both doses of Se caused the accumulation of this micronutrient in mice brain and liver and have also provided a disrupting effect on the levels of iron. Both doses of Se have triggered oxidative liver damage. In the brain, the effect of Se was dose dependent, where −0.2 mg of Se provided antioxidant activity, which was observed through a decrease in lipid peroxidation. On the contrary, the 0.4 mg dose increased brain catalase activity as well as gene expression, which may have contributed to maintaining brain lipid peroxidation at the control level.

## 1. Introduction

Selenium is an essential microelement for humans and animals. It is mainly taken up from the diet or the supplements of both organic (e.g., selenomethionine, selenocysteine) and inorganic (e.g., selenites, selenates) origin [1]. The form of entry into the body, its concentration, and its interaction with other elements in food determine the further metabolism of Se in the body [2,3]. It is known that the range of selenium intake between insufficient and toxic levels is very narrow, so it is important to carefully control the intake of this element [4]. The World Health Organization recommends a daily dose of Se at 55 µg for the adults [5]. Prolonged deficiency in or excess of Se causes a variety of disorders in the body. It can disrupt the immune system; may cause susceptibility to bacterial and viral infections; and increases the risk of mental, neurological, and cardiovascular disorders, as well as diabetes, endemic osteoarthropathy (Kashin–Beck disease), male infertility, cancers, and many others [6,7,8,9,10,11,12,13,14]. Although the body accumulates extremely small amounts of Se, being a component of at least 25 selenium-containing proteins (selenoproteins) in humans and 24 homologues proteins in rodents, it is vital for the maintenance of various, different body functions, such as regulation of oxidative stress, involvement in redox mechanisms, and other crucial cellular processes [15,16]. The liver is a major organ involved in the regulation of Se metabolism and maintenance of its level in the body [1]. Most of the hepatic selenium is used for the synthesis of selenoproteins [17]. One such a protein—selenoprotein P—enters the bloodstream from the liver, reaches the extrahepatic tissues, thus supplying cells with selenium [18]. Selenium also is particularly important in normal brain function maintenance, as it plays a vital role sustaining motor activity, cognition, coordination, and memory [19]. The entry of selenium into nerve tissue must be strictly controlled to avoid its toxicity due the excess, while its deficiency in the brain usually does not occur, even if its blood levels are low [20]. 

When received in adequate doses, Se, mainly in the form of selenocysteine, is inserted into the structure of antioxidant selenoproteins and exerts its antioxidant activity [21]. Meanwhile, at supranutritional doses, organic selenomethionine, as well as inorganic Se, can be toxic, thus revealing its prooxidant activity, causing an increased risk of brain disorders, endocrine system disruption, and cancer [6,22]. Excessive Se levels are known to generate reactive oxygen species (ROS), which subsequently disrupt the redox state of cells [23], resulting in increased oxidative stress and damage to organs and tissues [24]. High levels of free radicals or ROS are known to directly initiate the chain peroxidation reactions of membrane lipids [25]; thus, the level of oxidative stress in cells can be determined by the changes in the amount of the final lipid peroxidation product malondialdehyde (MDA), as well as by the alterations in the antioxidant enzyme activity and gene expression.

Laboratory mice provide ideal animal models for biomedical research and comparative medicine studies because they have many similarities to humans in terms of anatomy and physiology. The mouse and human genomes are about 85 percent the same and the principles of how genes are controlled are similar between the species, this makes the mouse an excellent system to model human physiology and pathophysiology [26,27]. Both Se excess and deficiency are associated with health risk in humans and mice [28].

The scientific literature emphasizing the effective and safe use of Se for prophylactic and therapeutic purposes are still insufficient and quite controversial, as different chemical forms of Se have quite diverse properties. Quite a number of studies, in various animal models, have been performed on the antioxidant effects of Se; however, the concentration limit at which the protective effect of Se (Na_2_SeO_3_) is altered to its prooxidant activity has not been fully investigated. To answer this question, in this study, we sought to evaluate the effect of different doses of Se on the development of oxidative stress in laboratory mice tissues, based on the changes in the content of malondialdehyde and alterations in antioxidant enzyme catalase activity and its gene expression. There are very limited data on the effects of Se excess on the other biogenic elements, such as iron (Fe) homeostasis, in mice brain and liver, which play a particularly important role in selenium metabolism. Disorder of iron homeostasis in the brain is known to affect neurophysiological mechanisms, cognition, and social behavior, and may therefore play an important role in the development of various neuropathological diseases [29]. Iron imbalance in cells is known to trigger prooxidant activity [30]; thus, the next objective of our study was to determine whether excess Se is associated with changes in Fe levels in the mouse brain and liver. 

## 2. Materials and Methods

### 2.1. Solvents and Reagents

All the standards, reagents, and solvents used throughout the experiments were of analytical grade. Na_2_SeO_3_, KCl, Tris, HCl, H_3_PO_4_, n-butanol, and 2-mercaptoethanol were from Sigma-Aldrich (Steinheim, Germany); TBA (thiobarbituric acid), sucrose, MgCl_2_, HNO_3_, and H_2_O_2_ were purchased from Serva (Heidelberg, Germany); Maxima SYBR Green/ROX qPCR Master Mix (2X), “Gene JET RNA purification kit”, and “High Capacity cDNA Reverse Transcription kit” were from Thermo Fisher Scientific (Waltham, MA, USA). 

### 2.2. Experimental Animals and Exposure Protocol

Experiments were performed with 4- to 6-weeks-old BALB/c white laboratory mice weighing 20–25 g. Mice were housed in a conventional animal room with a 12 h light/dark cycle. Mice were randomly assigned into three groups; one of them was the control group. Mice were given tap water supplemented with Na_2_SeO_3_ salt day-after-day for 8 weeks. The first group of mice were given tap water with a 0.2 Se dose (0.2 mg Se/kg body weight (BW)). The second group mice were given tap water with a 0.4 Se dose (0.4 mg Se/kg body weight). The control group mice had free access to tap water. Each group consisted of eight animals. Mice of all groups had been weighed once a week for evaluation of body weight gain. After an 8-week period, animals were anaesthetized, terminated, and their organs were removed. All the procedures were performed, and mice tissues handled in strict accordance with the rules defined by the European convention for the protection of vertebrate animals used for experimental and other scientific purposes (Republic of Lithuania, License of State Veterinary Service for working with laboratory animals No G2-80).

### 2.3. Determination of Malondialdehyde Content

The lipid peroxidation marker malondialdehyde (MDA) content in biological samples were measured as described by Uchiyama et al. using the thiobarbituric acid (TBA) test [31]. The assay is based on an MDA reaction with TBA producing red MDA-TBA2 adducts. Mice organs were homogenized in cold 1.15% KCl solution to make a 10% homogenate. Samples were prepared in centrifuging tubes containing a 3 mL 1% H_3_PO_4_, 0.5 mL tissue homogenate, and 1 mL 0.6% thiobarbituric acid aqueous solution. The prepared mixture was heated in a boiling water bath for 45 min. After the samples were cooled in ice bath for 10 min, 4 mL of n-butanol was added and mixed vigorously. The butanol phase was separated by centrifugation Beckman J2-21 (Beckman Instruments, Palo Alto, CA, USA) and absorbance was measured spectrophotometrically (UV/Vis spectrophotometer LAMBDA 25, (Perkin Elmer, Waltham, MA, USA) at 535 and 520 nm. The MDA concentration was expressed as nmol/g of wet organ weight.

### 2.4. Determination of Se and Fe Concentrations

The concentrations of Se and Fe in the brain and liver were evaluated by using an inductively coupled plasma mass spectrometer NexION 300 D (Perkin Elmer, Waltham, MA, USA) after microwave-assisted acid digestion. Samples were prepared in glassware that consist of 1 mL 69% HNO_3_, 1 mL 32% H_2_O_2_, 100 mg of biological sample, and 5 mL H_2_O. The glassware was placed in a microwave system Anton Paar Multiwave 3000 (Graz, Austria). By gradually increasing the pressure and temperature, the samples were digested. After digestion of the samples, they were diluted with deionized ultrapure water. This solution was finally used for elemental analysis using an ICP-MS system. To ensure the accuracy of the analysis, we conducted internal and external quality control procedures, including the use of analytical high-purity water and reagents. We also conducted the control of labware for contamination with trace elements. The concentrations of Se and Fe were expressed as µg/g in organs.

### 2.5. The Brain and Liver Homogenates Preparation

Following cervical dislocation of the animal, the brain and liver were removed, washed, and immediately cooled on ice. The organs were carefully weighed and homogenized in three volumes (relative to organ weight) of buffer (50 mM Tris-HCl, pH 7.6; 250 mM sucrose; 60 mM KCl; 5 mM MgCl_2_; 10 mM 2-mercaptoethanol). The homogenate was centrifuged at 15,000× *g* for 15 min centrifuge Beckman J2-21 (Beckman Instruments, Palo Alto, CA, USA)), and then postmitochondrial supernatant was used for the measurement of enzymatic activity in the organ tissues.

### 2.6. Protein Concentration Assay

The protein concentration in homogenate samples of the brain and liver was measured by using the Lowry method [32]. 

### 2.7. Measurement of Enzyme Catalase Activity 

Catalase (CAT) activity in the brain and liver homogenates was evaluated according to the method described in [33]. CAT activity was measured by hydrogen peroxide reaction with ammonium molybdate, which produces a complex that absorbs at a 410 nm light wavelength. The results are expressed in U/mg protein. Under these conditions, one unit of catalase (U) decomposes 1 μmol of hydrogen peroxide per minute.

### 2.8. Determination of Catalase Gene Expression

Catalase (CAT) gene expression was evaluated using a Real-Time PCR system (7500 Fast Real-Time PCR System (Applied Biosystems, Thermo Fisher Scientific, Waltham, MA, USA) and Maxima SYBR Green/ROX qPCR Master Mix (2X). The primer sequences for catalase gene were forward 5′AAGATTGCCTTCTCCGGGTG3′ and reverse 5′GACATCAGGTCTCTGCGAGG3′. The GAPDH gene was used as endogenous control for results normalization.

Mice brain and liver samples were digested using liquid nitrogen. After digestion, total RNA was extracted using the “Gene JET RNA purification kit”. In order to minimize the nonspecific amplification in real-time PCR reactions, the purity and quantity of extracted RNA was measured spectrophotometrically (spectrophotometer Nanodrop 2000, Thermo Fisher Scientific, Waltham, MA, USA). cDNA was synthesized using the “High Capacity cDNA Reverse Transcription kit”. 

The RT-PCR assay was performed using a 12 µL single-reaction combination consisting of 6 µL SYBR Green/ROX qPCR Master Mix 2X, 1 µL of both the forward and reverse primers, 3 µL 5 ng/µL cDNA, and 2 µL of nuclease-free water. The thermal cycling was performed as it adheres to the following: DNA denaturation at 95 °C for 10 min complied with 40 amplification cycles (95 °C 15 s, 60 °C 30 s and 72 °C 30 s).

The PCR reactions were kept on track by determining the strength of the fluorescence brought on by the SYBR Green Dye intercalation with double-stranded DNA. RT-PCR data were analyzed by the ΔΔCt method. The assay allows to calculate the change in relative gene expression (fold change), which determines the change between the final and initial values. The analysis is performed using Ct (cycle threshold) values, defined as the number of cycles required for the fluorescent signal to cross the threshold.

### 2.9. Statistical Analysis

Statistical analysis was performed using the statistical software package IBM SPSS Statistics (1.0.0.1416 version). Results are expressed as the mean ± standard error of the mean (SEM). The data were analyzed by a nonparametric Kruskal–Wallis test. Statistical significance was set at *p* ˂ 0.05. Graphical analysis of the results was performed with the MS Excel (2019) computer program.

## 3. Results

This study was performed in order to assess the levels of Se and Fe in the brain and liver of mice, as well as to determine the effects of different doses of Se on lipid peroxidation and antioxidant enzyme catalase activity, as well as its gene expression in the abovementioned organs after 8 weeks of mice exposure to Na_2_SeO_3_ solutions.

### 3.1. Evaluation of Selenium Effect on Body Weight and Relative Organ Mass Index of Mice

**Figure 1:**
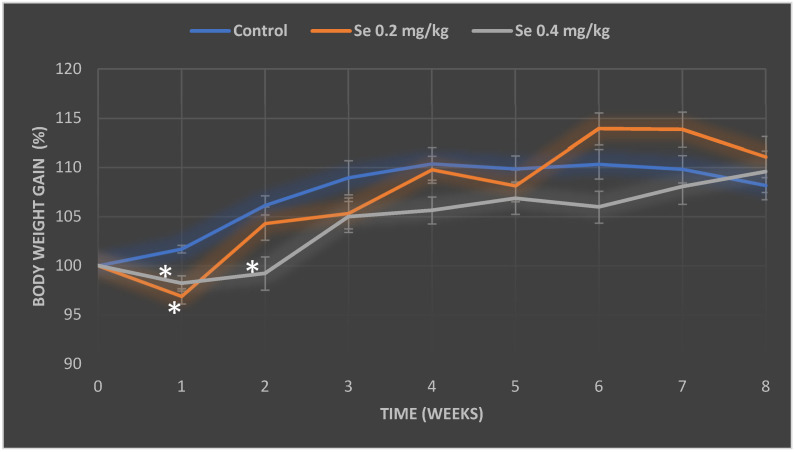
Time course of body weight gain of the control group mice and the mice orally treated with Na_2_SeO_3_ solution for 8 weeks. The medium weight gain in the groups was expressed as a percentage, and the initial weight of the mice in each group was equated to 100%. The model of selenium exposure to mice is described in the Methods section. The data were obtained by measuring the body weight of 16 mice in each group. *—differences are statistically significant in comparison to the control group; *p* ˂ 0.05.

To reach the goal, we chose to measure the changes in mouse body weight over an eight-week period and to estimate the relative organ mass indexes (ratio of organ mass to body weight). In toxicological studies, body weight and relative organ mass are considered as important criteria for the assessment of toxicity [34]. Evaluation of the changes in body weight of the control mice has demonstrated a steady increase of their weight during the first three weeks of the study; then, the growth has slowed down and remained at a similar level until the end of the experiment (Figure 1). Analysis of body weight gain tendencies in the experimental groups of mice, showed that different doses of Se did not have a significant effect on body weight gain (Figure 1). Analysis of weight gain tendencies in the experimental (selenium-treated) groups of mice showed no impact on body weight gain (Figure 1). On the contrary, a higher dose of selenium-treated mice had lower weights than control mice throughout the whole experiment; only for the last two weeks period, the weight of mice exposed to 0.4 mg/kg BW of Se have reached the level of the control. By the end of the second week, the weight gain of mice treated with a higher dose of Se (0.4 mg/kg BW of Se) had decreased by 6.9 percent (*p* < 0.05); however, in the subsequent weeks of the study this effect was no longer statistically significant compared to the body weight gain of control mice. The weight gain of mice treated with the lower dose of Se (0.2 mg/kg BW of Se) was similar to that of control mice, while in the final weeks of the study their weight gain has even exceeded the control. However, our observed body weight gain differences between the control and 0.2 mg Se-treated mice have showed statistical significance (4.8% decrease (*p* < 0.05)) only for the first week of the experiment; later they became negligible. Summarization of the final weight gain of mice have showed that, after an 8-week period, the highest weight gain (an increase of 11.1%) was observed among mice exposed to 0.2 mg of Se; the second highest weight gain (an increase of 9.6%) was spotted in group of mice that received the dose of 0.4 mg of Se, whereas the lowest weight gain (of 8.2%) was observed in the control mice group (Figure 1). 

**Figure 2:**
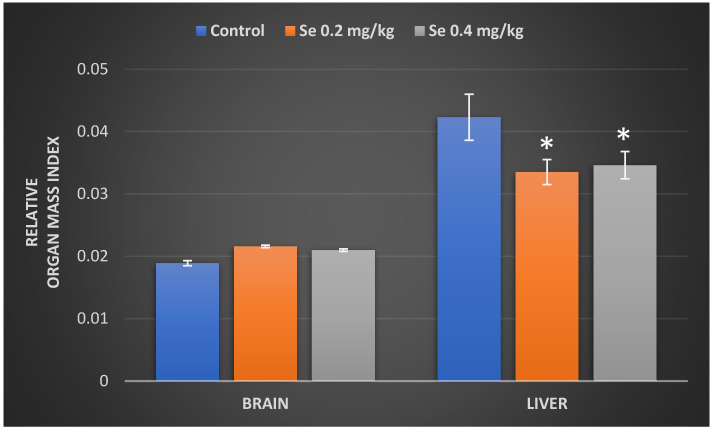
Relative mass index of the mice brain and liver (g of organ mass/g of body weight) of control group mice and mice orally treated with a Na_2_SeO_3_ solution for 8 weeks. The model of selenium exposure to mice is described in the Methods section. The data were obtained by measuring the body weight and organ mass of 16 mice in each group. Results were expressed as the mean ± SEM. *—differences are statistically significant in comparison to the control group; *p* ˂ 0.05.

The changes in organ mass are known as one of the most sensitive indicators of chemically induced organ damage. Therefore, the relative mass index of mouse brain and liver was evaluated after 8 weeks of exposure to Na_2_SeO_3_. The data in Figure 2 demonstrate that both the 0.2 and 0.4 mg/kg BW of Se doses have reduced the liver mass index; however, they had no significant effect on the mass index of the brain. Mice treated with the 0.2 and 0.4 mg/kg BW of Se doses demonstrated a statistically significant reduction in the relative liver mass index by 20.8% and 18.2%, respectively, as compared to the control. By the end of experimental period, a tendency of an increase (11–14%) in the relative brain mass index of Se-treated mice was observed; however, this change was not statistically significant.

### 3.2. Evaluation of Selenium and Iron Concentrations in Mice Organs

Results presented in Table 1 revealed that the Se amount is relatively low in mice brain after 8 weeks of oral consumption of an inorganic Na_2_SeO_3_ solution. The concentration of this element in control mice brain was 0.061 ± 0.012 µg/g. We found that the highest amount of Se accumulated in the brain of mice treated with a 0.4 mg/kg BW of Se dose. In this group, the Se concentration was 2.5-fold higher than the control and reached 0.154 ± 0.015 µg/g (*p* ˂ 0.05). However, the brain of mice treated with a 0.2 mg/kg BW dose of Se also accumulated significant amounts of this micronutrient, which was only slightly lower (0.145 ± 0.013 µg/g, *p* ˂ 0.05) than that of the 0.4 mg/kg BW Se-treated mice group. Our results also demonstrated that a 0.2 mg/kg BW dose of Se caused a statistically significant increase (by 15%) in the brain Fe concentration (32.40 ± 1.301 mg/g), and exposure to a 0.4 mg/kg BW dose of Se caused an even higher 20% increase in the Fe levels of the brain (32.73 ± 1.512 mg/g) as compared to the control group. Our experimental data demonstrates that the Se concentration in the liver of control mice was 0.615 ± 0.095 µg/g, while the exposure to Na_2_SeO_3_ has caused a statistically significant increase in the Se amount in mice liver regarding the control. The amount of selenium accumulated in the liver of mice treated with the 0.2 Se dose was 215% higher than its amount found in the control group. The accumulation of Se in the liver of mice treated with the 0.4 Se dose increased even further (exceeded the control by 240%) and have reached 2.11 ± 0.045 µg/g. Mice administration with the 0.2 mg/kg BW dose of Se has increased their Fe concentration in the liver by 112% (252.67 ± 10.613 mg/g, *p* ˂ 0.05), while exposure to 0.4 mg/kg BW Se dose has an even bigger impact, it increased the liver Fe amount (from 119.07 ± 11.259 to 275.09 ± 16.634 mg/g, *p* < 0.05) by 131%, which was 2.3-fold higher than that of the control mice. 

### 3.3. Evaluation of Malondialdehyde Content in Mice Organs after Exposure to Selenium

**Figure 3:**
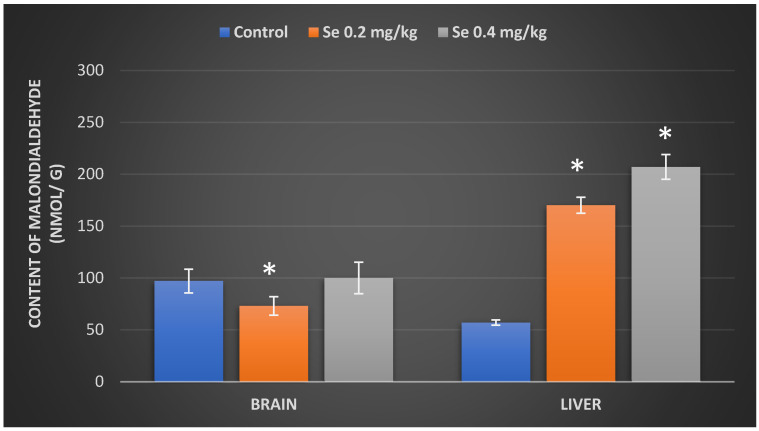
Content of malondialdehyde in mice brain and liver of the control group mice and mice orally treated with a Na_2_SeO_3_ solution for 8 weeks. The model of selenium exposure to mice is described in the Methods section. Data represent the results of eight separate experiments. Results are expressed as the mean ± SEM. *—differences are statistically significant in comparison to the control group; *p* < 0.05.

In order to evaluate the impact of inorganic Se excess on the tissue oxidative damage, in the next stage of our research, we sought to evaluate the effect of different Se exposure doses on the lipid peroxidation, using the final lipid peroxidation product malondialdehyde (MDA) as a marker of polyunsaturated acid oxidation. The results, represented in Figure 3, demonstrate that the treatment with a 0.2 mg/kg BW Se dose for 8 weeks resulted in a statistically significant (25%) reduction in mice brain MDA levels (73 ± 7.64 nmol/g) regarding the control (97 ± 2.56 nmol/g) group of mice; exposure to the 0.4 mg/kg BW dose of Se, however, had no effect on the content of mice brain MDA. In the liver of mice, both treatment with the 0.2 mg/kg BW and 0.4 mg/kg BW Se doses resulted in statistically significant growth in the MDA levels by, respectively, 198% (170 ± 13.76 nmol/g) and 263% (207 ± 14.87 nmol/g) as compared to the control group of mice.

### 3.4. Determination of Catalase Activity and CAT Gene Expression in Mouse Organs after Exposure to Selenium

**Figure 4:**
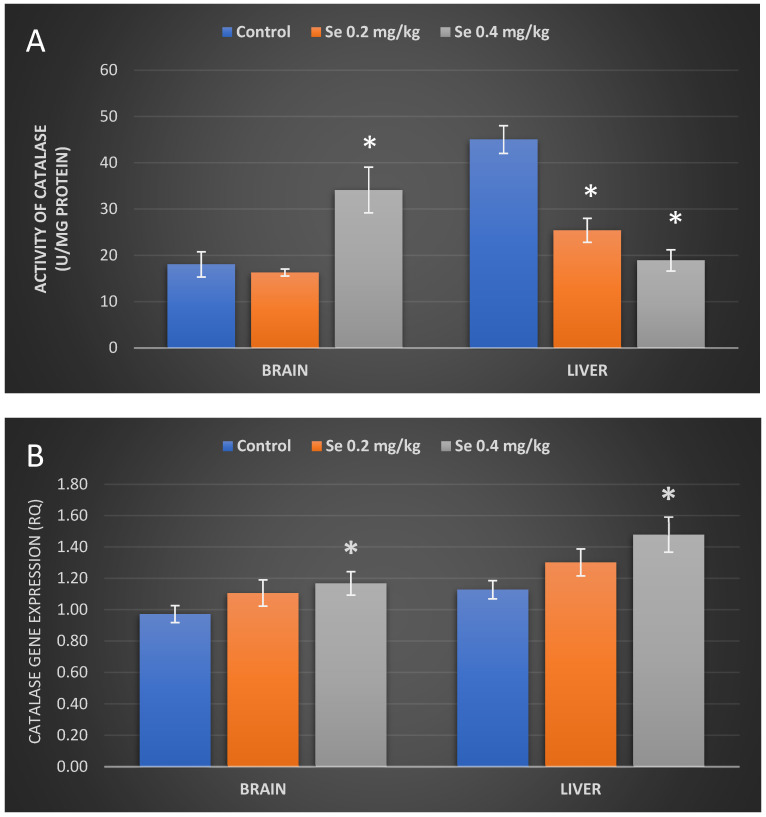
Activity of catalase (**A**) and relative expression of CAT gene (**B**) in mice brain and liver of the control group mice and mice orally treated with a Na_2_SeO_3_ solution for 8 weeks. The model of selenium exposure to mice is described in the Methods section. Data represent the results of eight separate experiments. Results are expressed as the mean ± SEM. *—differences are statistically significant in comparison to the control group; *p* < 0.05.

Data that represent the effects of Se on antioxidant enzyme catalase activity in mouse organs are showed in Figure 4. Brain catalase activity in mice treated with the 0.2 mg/kg BW Se dose remained almost at control levels and have reached 16.25 ± 0.69 U/mg protein (Figure 4A); in addition, no significant differences from the control were observed in brain CAT gene expression as well (Figure 4B). On the contrary, a 0.4 mg/kg BW dose of Se provoked a significant increase (by 20%) in brain CAT gene expression (Figure 4B) and statistically significantly raised the brain catalase activity by 89% (34.08 ± 4.42 U/mg protein) (Figure 4A). Our following results showed that the liver catalase activity of mice treated with a 0.2 mg/kg BW dose of Se was 25.4 ± 2.36 U/mg protein and was statistically significantly lower (by 44%) than that of the control (45 ± 2.75 U/mg protein) group of mice (Figure 4A). However, no significant differences in liver CAT gene expression in mice treated with the 0.2 mg/kg BW dose of Se were observed (Figure 4B). The 0.4 Se dose has suppressed liver catalase activity by 58% (18.9 ± 2.12 U/mg protein) compared to controls; however, it statistically significantly increased CAT gene expression by 31% in the liver of BALB/c mice (Figure 4B).

## 4. Discussion

The aim of our study was to evaluate the effect of selenium excess on the oxidative stress level in BALB/c mice brain and liver. Doses of selenium in our experimental studies were selected based on the data provided in scientific sources. The recommended daily dose of Se for men and women is set at 55 μg. Scientific data suggest that the tolerable maximum daily intake of Se is of 400 µg; when daily intake is regularly greater than 400 µg, the adverse effects of selenosis are expected to occur [35]. Experimental data show that the therapeutic and antioxidant effects of Se occur at doses several times higher than those required to avoid clinical signs of Se deficiency, while the anticancer properties of Se occur in rodents at doses of 100–500 μg Se/kg BW per day [36]. In our study, the amount of Se that laboratory mice daily received with drinking water (in the form of Na_2_SeO_3_) was 2-fold (0.2 mg Se/kg BW) and 4-fold (0.4 mg Se/kg BW) higher than the dose of Se recommended for laboratory mice (0.1 mg/kg) per day [37].

Selenium is a trace element required for the normal functioning of various human and animal systems, especially those of nervous, immune, reproductive, cardiovascular systems [6]. However, Se is known to affect the whole organism; thus, at first, we have evaluated the total systemic effect of Na_2_SeO_3_ on the changes of mouse body weight as well as the effect on the relative organ mass index over the period of 8 weeks. The adequate intake of Se is essential for the growth, proper functioning of the immune system [38], and for a great variety of others biochemical–physiological functions [39]. Its deficiency is associated with disorders of bone metabolism [40] and thyroid hormone regulation [41].

In our study, all BALB/c mice have survived to the end of the experiment. The results we obtained did not show any growth-promoting effect of Se in laboratory mice after 8 weeks of supplemental administration of Na_2_SeO_3_. At the end of our experiment, the total weight gain of the mice was quite similar: the highest increase (11.1%) in the body weight was seen in the mice group treated with 0.2 mg Se/kg BW, the second highest (9.6%) body weight increase was observed in the group of mice treated with 0.4 mg Se/kg BW, and the lowest body weight gain (8.2%) was seen in the control group mice; however, these differences were not statistically significant. Our results are consistent with those reported by other researchers in studies with BALB/c mice exposed to 0.045, 0.1, 0.4, and 0.8 mg Se/kg BW per day for a period of 56 days [15]. Data provided by the other group of scientists showed a growth-promoting effect of Se in male mice, with a 20 μmol/L Na_2_SeO_3_ dose and the duration of the experiment being twice as long [42]. Another study performed with rats showed that for the animals fed a diet containing 5 μg of Se (Na_2_SeO_3_) per 1 g of feed, the gain of weight began to slow down from the 10th day of the experiment and continued to decline until the end of the experiment (28 days) as compared to rats that received significantly lower Se intakes with a feed (0.08, 0.24, 0.8 μg Se/1 g feed) [43].

The results of our experiment also showed that mice, daily administrated with 0.2 and 0.4 mg Se/kg BW in drinking water, showed a decrease in the index of their liver mass. The obtained reduction in liver mass index may be related to the histological and ultrastructural changes reported by Tos-Luty and co-authors [44]. The researchers stated that sodium selenite supplementation in a lower dose (0.5 mg/kg BW) for 10 days caused microfocal infiltrations in the liver. In the ultrastructure of hepatocytes, widened ergastoplasm canals, single lipid drops, and shortened crista of mitochondria were observed. A higher sodium selenite dose (1 mg/kg BW) resulted in infiltrations of mononulear cells, and the inflammation was accompanied by granulocytes and focal hepatocyte degeneration [44]. In contrast, Zhang and co-authors [15] have reported an increase in mass indexes of mice liver and kidney at doses of 0.4 mg Se/kg, and some others as well. 

In the next stage of our study, we have determined the concentrations of Se in the brain and liver of BALB/c mice. Our results showed the highest amount of Se accumulates in the liver within the period of 8 weeks. We found that long-term consumption of sodium selenite significantly increased the liver levels of Se more than 3-fold while the brain Se levels—2.5-fold. However, in an experiment of Zhang et al. [15], where BALB/c mice for 56 days were fed a Se supplemented feed, of the same 0.4 mg Se/kg BW dose, concentration of Se in mice liver significantly differed from the concentration of Se in the liver of our experimental mice, which showed up as almost 1.6-fold higher compared to the mice liver Se levels in Zhang and co-workers’ designed experiment. These results are consistent with studies published by other researchers on the accumulation of Se in the liver of various organisms (including rodents), and elevation of its levels in the blood [15,45,46]. The liver is known to be the main regulator of the amount of Se in the whole body; it does it by synthesizing selenoproteins, which are then secreted into the tissues to maintain the body Se levels in the normal ranges [1]. The scientific data indicates that, in the presence of excess Se in the body, the liver increases its elimination, thus only slightly increasing its content in other organs [1].

Our results showed that the concentration of Se in the brain, as compared to other organs studied, is relatively low (0.061 ± 0.012 µg/g); however, mice that were treated with Se in drinking water accumulated significantly higher brain Se levels than did the control group mice. We found that highest levels of Se was accumulated in the brain of mice exposed to the 0.4 mg Se/kg BW dose, where it was 2.5-fold higher compared to the control. However, brains of mice treated with the 0.2 mg/kg BW Se dose had also accumulated quite high levels of Se, which were only slightly lower than those treated with the 0.4 mg Se/kg BW dose. Although, there are some scientific data stating that the amount of Se in the brain and cerebrospinal fluid is independent of its blood levels, as the brain Se levels are tightly regulated by the blood–brain barrier in order to protect the nervous tissue from Se deficiency as well as its toxicity [20,47]; however, our experimental data showed that, in the case of Se excess, its concentration in the brain significantly increases. In summary, we can state that under the conditions of our experiment, the livers of selenium treated mice not only accumulated large amounts of Se but also did not prevent the increase of Se in the brain. We also observed that livers of selenium-treated mice showed impairment in iron homeostasis. This parenchymal organ as treated with 0.2 and 0.4 mg Se/kg BW doses for 8 weeks, had accumulated, respectively, 2.1-fold and 2.3-fold higher Fe levels than the control mice. Iron plays a key role in most physiological processes, and its disruption of homeostasis has a major impact on many organs, and the brain is particularly sensitive to such changes [31].

To assess whether high levels of Se accumulated in mice tissues may contribute to the development of cellular oxidative damage, we have evaluated the effect of different doses of Se on the formation of malondialdehyde. Being one of the end products of polyunsaturated fatty acid peroxidation in the cell, MDA is an important marker of oxidative lipid damage [25]. Results of our study showed, that selected doses of Se had indeed increased formation of reactive oxygen species and activated lipid peroxidation in the liver of selenium-treated mice. Similar results were reported by Zhang and co-authors [15]. Our results are consistent with other scientific data showing that at high doses Se tends to induce apoptosis, causes oxidation of glutathione (GSH) and other cellular thiols, disturbs cellular redox status, thus increasing ROS generation [48]. Accumulated in cells excess of Se reacts with reduced glutathione to form highly active selenopersulfide [49], which then further reacts with a new molecule of GSH, thus forming the superoxide anion. The prooxidant properties of Se are generally associated with inorganic selenite, whereas selenomethionine and selenocysteine, especially their L-isoforms, are known to be less toxic [50].

Our obtained results are not sufficient to suggest that the prooxidant Se effect in the liver is direct. The observed prooxidant activity may be a consequence of the disruption of homeostasis of other elements, such as Fe. Our data showed that exposure to selenium significantly increased the concentration of Fe in the liver of mice as well as the expression of the superoxide dismutase gene (unpublished data), which suggests that the formation of ROS is indeed increased. On the other hand, the 0.2 mg Se/kg BW dose suppressed lipid peroxidation in the brain, which suggests that Se may have exerted an antioxidant effect in this organ. The antioxidant effect of selenium is likely due to its importance as a coenzyme in the activity of antioxidant system enzymes, such as glutathione peroxidase.

In the next step of our study, we sought to assess whether the Se-induced lipid peroxidation, observed in our study, was somehow related to the activity of an antioxidant system enzyme catalase, which does not depend on Se as a cofactor. Scientific evidence suggests that excess Se increases both the amount of lipid peroxides as well as the amount of hydrogen peroxide (H_2_O_2_), which is a substrate for catalase [48]. Catalase then breaks down H_2_O_2_ molecules into oxygen and water, thus preventing them from accumulating in cells and causing oxidative damage [24]. The literature indicates that the highest levels of catalase are found in the liver [24], red blood cells, and kidneys [51]. The results of our study have demonstrated that catalase activity in the liver of Se-treated mice was almost twice as low as compared with enzymatic activity of the control mice group. Although one of the main functions of the liver is detoxification and neutralization of various harmful substances, in order to protect other tissues from the toxic effects of these compounds [52], the detoxifying capacity of the liver has been shown to be insufficient under our experimental conditions. Active expression of the CAT gene suggests that liver cells produce significant amounts of hydrogen peroxide. Although H_2_O_2_ itself is not a radical, it is capable of oxidizing various cofactor metal ions, protein -SH groups, or Fe-S centers, thereby causing additional iron release into the cell. An increased amount of metal ions in the cell activates the reactions of Fenton or Haber–Weiss, thus leading to H_2_O_2_ conversion to hydroxide radicals (OH•). Particularly, active OH• has been shown to damage proteins, DNA, and lipids [53,54]; it may also inhibit catalase synthesis or disturb regulation of its activity [55]. The effect of ROS on various proteins, including the enzyme catalase itself, can be threefold: ROS oxidizes amino acid residues, causes hydrolysis of peptide bonds, or may induce aggregation of the proteins themselves [56]. Catalase activity has been shown to be inhibited by a variety of compounds, including its natural substrate H_2_O_2_ (above 0.1 mol) [57]. Increased levels of MDA can also suppress its activity [25]. Thus, there may have been quite a few reasons that caused inhibition of the liver catalase activity observed in our study.

However, we have not noticed any catalase enzymatic activity changes in the brain of mice exposed to the 0.2 mg Se/kg BW dose, although the lipid peroxidation levels in brain tissue were significantly reduced. This suggests that the selenium concentration in the brain of the 0.2 mg Se dose-treated mice was favorable for the antioxidant activity of selenoproteins such as glutathione peroxidase to occur, thus protecting against oxidative damage. Mice for 8 weeks exposed to the 0.4 mg Se/kg BW dose have showed a statistically significant increase in both CAT gene expression and catalase activity in the brain, while lipid peroxidation remained at the level of the control group of mice. Perhaps a higher dose of Se led to its increased accumulation, resulting in a more pronounced prooxidant effect, which then results in increased catalase activity. Similar results on catalase activity in the brain have been demonstrated by Agarwal and Behari [58], although more in-depth studies are needed to completely understand the capability of selenium to behave as an anti/prooxidant agent.

## 5. Conclusions

In mice, an 8-week oral treatment with inorganic a Na_2_SeO_3_ solution in 0.2 mg/kg BW and 0.4 mg/kg BW Se doses resulted in a relatively low accumulation of Se in the brain of mice; however, it induced a 10-fold increase in its accumulation in the liver of mice. Exposure to both doses of selenium resulted in impaired iron homeostasis and increased iron accumulation in the tested organs. Both doses of Se have triggered oxidative liver damage, which was observed through the increased lipid peroxidation as well as decreased catalase activity, while the 0.4 mg/kg BW Se dose has also activated catalase gene expression. In the brain of mice, a 0.2 mg/kg BW dose of Se has showed some antioxidant activity that was observed through a decrease of lipid peroxidation and no change in catalase activity. On the contrary, a 0.4 mg/kg BW dose of Se increased the brain catalase activity as well as gene expression, which may have contributed to maintaining brain lipid peroxidation at the control level.

## Figures and Tables

**Table 1:** Concentrations of selenium and iron in the brain and liver of control group mice and mice orally treated with a Na_2_SeO_3_ solution for 8 weeks. The model of selenium exposure to mice is described in the Methods section. Data represent the results of eight separate experiments. Results are expressed as the mean ± SEM. *—differences are statistically significant in comparison to the control group; *p* ˂ 0.05.

Mice Group	Concentration of Selenium	
	Brain (µg/g)	Liver (µg/g)
Control	0.061 ± 0.012	0.615 ± 0.095
Se 0.2 mg/kg BW	0.145 ± 0.013 *	1.946 ± 0.069 *
Se 0.4 mg/kg BW	0.154 ± 0.015 *	2.108 ± 0.045 *
	**Concentration of Iron**	
	**Brain (µg/g)**	**Liver (µg/g)**
Control	27.265 ± 1.895	119.068 ± 11.259
Se 0.2 mg/kg BW	32.404 ± 1.301 *	252.673 ± 10.613 *
Se 0.4 mg/kg BW	32.731 ± 1.512 *	275.093 ± 10.613 *

## Data Availability

Data is contained within the article.
